# Perioperative outcomes and tissue trauma after uncomplicated total hysterectomy for benign uterine disease: a retrospective study

**DOI:** 10.3389/fsurg.2026.1877169

**Published:** 2026-10-23

**Authors:** Corrado Terranova, Andi Stermasi, Roberto Montera, Carlo De Cicco Nardone, Daniela Luvero, Federica Guzzo, Gianna Barbara Cundari, Adele Silvagni, Arianna Martinelli, Erika Notaro, Nicola Alboré, Roberto Angioli, Francesco Plotti

**Affiliations:** 1 Department of Gynecology, Fondazione Policlinico Universitario Campus Bio-Medico, Rome, Italy; 2 Research Unit of Gynecology Oncology, Department of Medicine and Surgery, Università Campus Bio-Medico di Roma, Roma, Italy; 3 Natl. Center for Radioprotection and Computational Physics, Istituto Superiore di Sanità, Roma, Italy; 4 Department of Physics, University of Rome “Tor Vergata”, Rome, Italy

**Keywords:** abdominal hysterectomy, benign uterine disease, laparoscopic hysterectomy, perioperative outcomes, systemic inflammation, vaginal hysterectomy

## Abstract

**Background:**

Hysterectomy is the most frequently performed non-obstetric gynecological surgery for benign disease, and the surgical route affects perioperative outcomes and recovery. While minimally invasive approaches reduce surgical morbidity, their relative effect on the systemic inflammatory response remains incompletely characterized, particularly when indication and case complexity are accounted for. We compared tissue trauma, operative data, and early postoperative outcomes after uncomplicated vaginal hysterectomy (VH), laparoscopic hysterectomy (LH), and abdominal hysterectomy (AH).

**Methods:**

We retrospectively reviewed women aged 25–80 years who underwent total hysterectomy for benign indications at our institution (2021–2024). Patients with malignant disease, chronic inflammatory conditions, prophylactic drains, or complicated postoperative courses were excluded. Primary endpoints were postoperative C-reactive protein (CRP) levels and white blood cell (WBC) and neutrophil (Neu) count trajectories over the first three postoperative days; secondary endpoints were hemoglobin drop, operative time, blood loss, bowel function recovery, and length of stay. Multivariable models adjusted for age, body mass index, American Society of Anesthesiologists (ASA) physical status, surgical indication, and previous abdominal surgery, with uterine weight assessed in a sensitivity analysis; trajectories were modelled with linear mixed-effects models, and the route effect was examined within a single-indication (fibromatosis) subgroup.

**Results:**

We included 281 patients (84 VH, 86 LH, 111 AH). AH and LH required longer operative times than VH, and AH was additionally associated with higher blood loss, delayed gastrointestinal recovery, and longer hospitalization. In unadjusted analyses AH showed the highest CRP at all three days and higher WBC and neutrophil counts on Days 1 and 2; the haematological differences were attenuated after adjustment, whereas the CRP elevation persisted. Because route, indication, and uterine size were strongly collinear, the route effect was examined within the fibromatosis subgroup, where AH remained independently associated with higher CRP than LH across all postoperative days.

**Conclusions:**

After uncomplicated hysterectomy for benign disease, surgical route was independently associated with the postoperative systemic inflammatory response. AH carried a greater inflammatory burden and slower functional recovery, whereas VH and LH were broadly comparable; compared with LH, VH showed faster recovery and certain perioperative advantages. Minimally invasive techniques should be prioritized when clinically feasible.

## 1 Introduction

Surveillance reports on hysterectomies performed in the United States have demonstrated a general decline in the overall number of procedures, decreasing from approximately 600,000 in 2003 to around 480,000 in 2009. Despite this reduction, benign indications have consistently accounted for the majority of cases exceeding 86.6%, reinforcing hysterectomy as the most commonly performed non-obstetric gynecological surgery (1, 2). The traditional abdominal hysterectomy (AH) and vaginal hysterectomy (VH) approaches have progressively been complemented by laparoscopic hysterectomy (LH), robot-assisted surgery, and hybrid techniques such as laparoscopic-assisted vaginal hysterectomy (LAVH) and the more recent transvaginal natural orifice transluminal endoscopic surgery. Despite the development of numerous novel technologies, VH, when feasible, remains the preferred approach due to its well-documented advantages and sustained benefits over alternative techniques (3, 4). Despite these well-established benefits, the use of VH has progressively declined in recent years, in favor of other minimally invasive techniques such as laparoscopy (5, 6). LH has undergone significant technological advancements and currently represents the most commonly employed approach. However, long-term data on complications associated with these two minimally invasive techniques remain inconclusive and may suggest comparable outcomes (5, 7). Regardless of the surgical access route employed, the release of inflammatory mediators following the procedure is inevitable. The magnitude of this cytokine-mediated response generally correlates closely with the extent of surgical trauma (8). This phenomenon has also been confirmed in gynecologic surgery, where it has been demonstrated that the inflammatory response triggered by AH is significantly greater than that induced by minimally invasive hysterectomy (9). Since the initial attempts to assess the surgical invasiveness of gynecologic procedures through serum biomarkers, numerous studies have sought to characterize and differentiate the inflammatory response according to the type of surgical access. The majority of these investigations have focused on comparisons between AH and LH. Nonetheless, conflicting evidence persists regarding the relative magnitude of inflammatory response between VH and LH. In this context, we aimed to evaluate whether the surgical route of hysterectomy is independently associated with postoperative systemic inflammatory response and perioperative outcomes in women undergoing uncomplicated hysterectomy for benign disease. Accordingly, rather than reassessing the established hierarchy of hysterectomy routes, this study sought to determine whether the three approaches are associated with distinct postoperative inflammatory trajectories after uncomplicated surgery, using serial routinely available biomarkers and analyses adjusted for clinical indication and case complexity.

## 2 Materials and methods

This single-center, retrospective observational study was conducted at the Fondazione Policlinico Universitario Campus Bio-Medico (Rome, Italy) and included consecutive women who underwent total hysterectomy for benign indications between January 2021 and December 2024. The study adhered to Good Clinical Practice and the principles of the Declaration of Helsinki and received approval from the Institutional Review Board (of Campus Bio-Medico of Rome, No 18/23 INT ComEtCBM, 251 date 8 March 2021). Written informed consent for the use of clinical data was obtained from all participants.

Inclusion and exclusion criteria are detailed below. Eligible patients were women aged 25–80 years who underwent total hysterectomy via VH, LH, or AH and for whom complete preoperative, intraoperative, and postoperative data were available. Exclusion criteria comprised hysterectomy performed for malignant indications; the presence of chronic inflammatory conditions potentially affecting baseline or postoperative inflammatory status (including deep infiltrating endometriosis, systemic lupus erythematosus, lymphomas, and chronic inflammatory bowel diseases); chronic or ongoing use of systemic anti-inflammatory or immunomodulatory medications; surgery performed under spinal or combined spinal–general anesthesia; and intraoperative placement of prophylactic surgical drainage. In case of postoperative complications, these were recorded and classified according to the Clavien-Dindo grading system.

As the primary aim of the study was to assess the systemic inflammatory profile following uncomplicated hysterectomies, patients who experienced a complicated postoperative course (Clavien-Dindo grade I or higher) were excluded from the statistical analysis. This exclusion was adopted to minimize clinical heterogeneity and to isolate the inflammatory response attributable to the surgical access route itself, avoiding confounding effects related to specific complication treatments such as reinterventions or prolonged antibiotic therapy.

Data were extracted from electronic medical records of patients discharged between 2021 and 2024 with procedure codes corresponding to total hysterectomy: 68.4 (total abdominal hysterectomy), 68.41 (laparoscopic total abdominal hysterectomy), 68.49 (other and unspecified total abdominal hysterectomy), 68.5 (vaginal hysterectomy), and 68.59 (other and unspecified vaginal hysterectomy). LAVH was excluded from the analysis, as the study was designed to compare perioperative outcomes among clearly distinct surgical routes without overlap between techniques. Consistent with this rationale, patients who required intraoperative conversion from the initially planned surgical approach were also excluded from the final analysis. All records meeting the inclusion and exclusion criteria were reviewed.

Baseline demographic variables collected included patient age, body mass index (BMI), and the American Society of Anesthesiologists (ASA) physical status classification. Surgical indications for hysterectomy were also collected and categorized. The choice of surgical approach was determined by preoperative anatomical and clinical criteria, including uterine size and mobility, vaginal accessibility, presence of pelvic organ prolapse, and prior abdominal surgery. All patients received the same preoperative preparation, consisting of intravenous administration of 2 grams of cefazolin prior to operating room entry. In patients with beta-lactam allergy, clindamycin 900 mg IV plus gentamicin 5 mg/kg IV was administered. No postoperative antibiotic doses were routinely given. A single dose of 4,000 IU of heparin was administered the evening before surgery. All procedures were performed under general anesthesia, as per the study exclusion criteria, with endotracheal intubation. All procedures were performed by a limited number of senior surgeons within the same surgical team, each experienced in VH, LH, and AH, without restriction to a specific approach, in order to minimize surgeon-related bias. Perioperative recorded observables included: Operative time [min], Estimated blood loss [mL], Days to postoperative bowel function recovery [day], Length of stay in hospital [day], Doses of analgesics [unit], and Time to first canalization of gas [day]. Estimated intraoperative blood loss was assessed according to a standardized institutional protocol. The volume collected in the suction canister was recorded after subtraction of the irrigation fluid used during the procedure. In addition, surgical sponges and gauzes were weighed, and blood loss from this source was calculated as the difference between wet and dry weight, assuming 1 gram to correspond to approximately 1 mL of blood. Minor blood loss absorbed by surgical instruments, drapes, or gowns was considered negligible and was not included in the calculation. Uterine weight was also included, as reported by the pathology department. Hemoglobin, WBC, and Neu measurements were performed at baseline and postoperatively at 24, 48, and 72 h, whereas CRP was assessed only at the three postoperative time points. Pain-management protocols were standardized across surgical routes, and postoperative rescue analgesia consisted of intravenous non-steroidal anti-inflammatory drugs (NSAIDs), recorded as the number of doses administered. The discharge criteria for all patients were: absence of fever during the preceding 24 h, a Numeric Rating Scale (NRS) pain score under 2, complete mobilization, stable hemoglobin levels, adequate spontaneous voiding following removal of the urinary catheter, and passage of flatus. Laboratory parameters were systematically collected preoperatively and on postoperative Days 1, 2, and 3. These included hemoglobin concentration (Hb, g/dL), total WBC count ($\times 10^{3}/\text{μ}L$), and absolute Neu count ($\times 10^{3}/\text{μ}L$), which were available both preoperatively and postoperatively. C-reactive protein (CRP) concentration (mg/dL) was routinely assessed only during the postoperative period, as it is not part of the standard preoperative laboratory evaluation for asymptomatic patients undergoing elective benign hysterectomy at our institution.

The primary outcomes of interest were postoperative CRP levels and perioperative trajectories of WBC and Neu counts on postoperative Days 1, 2, and 3 after surgery across the three surgical approaches. Because preoperative CRP was not routinely measured, CRP was analyzed as postoperative levels rather than as change from baseline. Secondary outcomes included hemoglobin drop, operative time, blood loss, hospital stay, bowel-function recovery time, and postoperative analgesic use.

Descriptive statistics were reported as n (%) for categorical variables, mean and standard deviation for demographic and perioperative variables with approximately normal distribution (assessed using the Shapiro–Wilk test), median with interquartile range (IQR) for all inflammatory markers pre and postoperative. Comparisons of demographic and clinical variables were performed between the three groups (AH, LH, and VH) using the Kruskal-Wallis test for continuous variables, and in cases where the global test was significant, post-hoc pairwise comparisons were conducted using the Mann-Whitney U test with Bonferroni correction to adjust for multiple testing. Categorical variables were compared using the chi-squared test of independence or Fisher’s exact test when sample size was small. A two-sided P-value of less than 0.05 was considered statistically significant for global tests.

Multivariable linear regression was used to evaluate the independent association between surgical approach and each postoperative inflammatory marker (CRP, neutrophil, and white blood cell count). CRP was log-transformed prior to modelling to address right skewness. All models used VH as the reference category and adjusted for age, BMI, ASA physical status, surgical indication, and previous abdominal surgery. Surgical indication was coded as a binary variable (prolapse vs. non-prolapse), as pelvic organ prolapse was the only indication distinguishing VH; the granular indications reported in Table 1 were collapsed accordingly for modelling. Because uterine weight could be retrieved for only 188 of 281 patients, and these missing measurements were non-random with respect to surgical route, uterine weight was not included in the primary models but was instead evaluated in a complete-case sensitivity analysis. As the three surgical routes corresponded to largely distinct clinical populations, we assessed multicollinearity using the variance inflation factor (VIF). Surgical route and indication were strongly collinear by design, since VH consisted almost entirely of prolapse cases and fibromatosis occurred exclusively in the laparoscopic and abdominal groups. To estimate the route effect within a clinically homogeneous population, we therefore performed a pre-specified stratified analysis restricted to patients operated for uterine fibromatosis, in which LH and AH could be compared directly (VH was not performed for this indication). To account for the repeated measurement of inflammatory markers within patients across the three postoperative days, we additionally fitted linear mixed-effects models with a patient-level random intercept, including a surgical route by postoperative day interaction to test whether marker trajectories differed by route. The significance of the interaction was assessed by a likelihood-ratio test comparing models with and without the interaction term, fitted by maximum likelihood. Day-specific regression models are additionally reported for interpretability and are provided in full in Tables 4–7. Analyses were performed in Python (v3.10.1) using SciPy (v1.15.2) (10) and statsmodels (v0.14.6).

**Table 1:** Patient characteristics stratified by route of hysterectomy.

Variables	VH	LH	AH	P-value
Num. of patients	84	86	111	
Age (years)	67.21±10.42	53.35±8.36	56.78±10.42	<0.001
BMI ($\mathrm{kg}/m^{2}$)	25.10±3.20	25.45±5.16	26.84±4.80	0.014
Uterine weights (g)	41.2 (31.0–65.5)	156.0 (95.8–250.3)	289.0 (76.3–684.1)	<0.001
Prior abd. surgeries	36 (42)	48 (55)	66 (60)	
ASA classes				0.27
1	7 (8.3)	2 (2.3)	4 (3.6)	
2	68 (81.0)	76 (88.4)	91 (82.0)	
3	9 (10.7)	4 (4.7)	10 (9.0)	
4	0 (0.0)	0 (0.0)	1 (0.9)	
Top surgical ind.				
1	${\text{POP}}^{*}$ (60)	Fibromatosis (30)	Fibromatosis (55)	
2	${\text{POP, SUI}}^{*}$ (37)	Hyperplasia (10)	Adenomyosis (7)	
Preoperative markers				
Hb (g/dL)	13.4 (12.7–14.2)	13.4 (12.4–14.1)	13.2 (11.9–13.9)	0.32
Neu ($\times 10^{3}/\text{μ}L$)	4.0 (3.3–5.2)	3.7 (3.1–5.1)	4.2 (3.2–5.2)	0.25
WBC ($\times 10^{3}/\text{μ}L$)	7.1 (5.9–8.2)	6.3 (5.5–7.9)	6.8 (5.7–7.8)	0.12

Data are presented as mean ± standard deviation or median (interquartile range), as appropriate, for continuous variables and n (%) for categorical variables. P-values were calculated using the Kruskal–Wallis test for continuous variables and the chi-squared test for categorical variables. * POP = Pelvic Organ Prolapse, SUI = Stress Urinary Incontinence, Fibromatosis refers to uterine leiomyoma (fibroids).

AH, abdominal hysterectomy; LH, laparoscopic hysterectomy; VH, vaginal hysterectomy; ASA, American Society of Anesthesiologists; BMI, body mass index; Hb, hemoglobin; Neu, neutrophils; WBC, white blood cells.

## 3 Results

According to the records obtained from the hospital administrative offices, a total of 560 procedures coded as total hysterectomies were performed during the study period (Figure 1). Of these, 33 were excluded as they had been carried out either by other surgical units or in consultation for external units, which did not adhere to the same postoperative management protocols. The remaining cases were subsequently screened according to predefined inclusion and exclusion criteria, resulting in the exclusion of 232 patients. Among the 295 eligible patients, 14 patients experienced postoperative complications and were therefore excluded from the statistical analysis (see Section 2).

**Figure 1:**
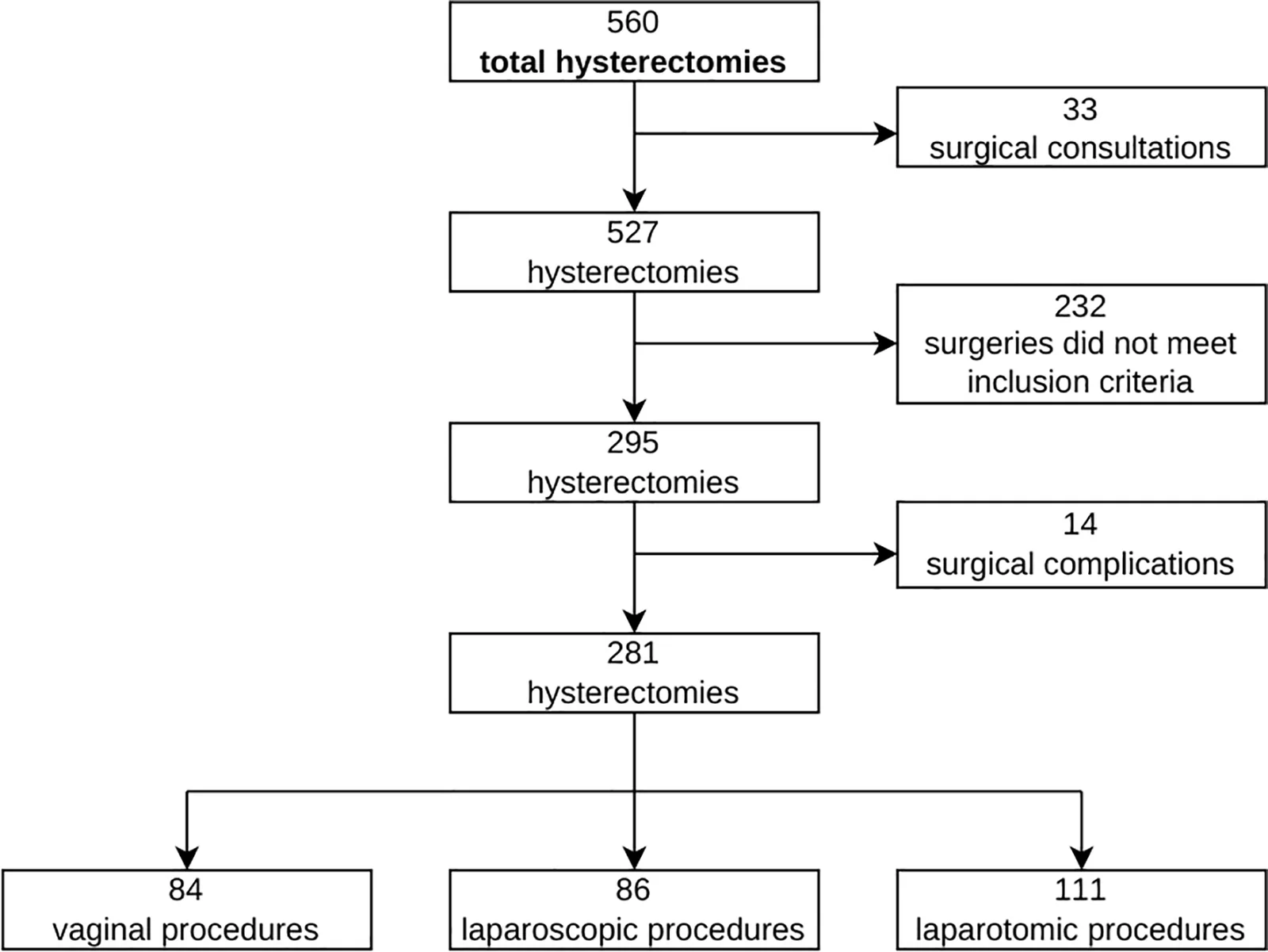
Flowchart of patients included in the study.

Among the 117 AH, complications occurred in 6 cases (5.13%). Specifically, three patients required blood transfusion and antibiotic therapy for abdominal wall hematomas, while three patients underwent reoperation under general anesthesia, two for postoperative pelvic hematomas with active bleeding, and one for acute diverticulitis requiring sigmoidectomy with colorectal anastomosis performed by the general surgery team. In the LH group, complications were observed in 4 of 90 cases (4.44%): one patient required a postoperative blood transfusion, one underwent reoperation under general anesthesia for postoperative bleeding, and two patients required surgical revision for ureteral injuries. In the VH group, the overall complications rate was 4.54% and no complications of grade III or higher were recorded. Two patients were treated with antibiotic therapy for vaginal cuff abscesses, one patient required a blood transfusion, and one patient required prolonged bladder catheterization due to vesical atony.

Demographics and perioperative characteristics of the patients admitted to the study are summarized in Table 1. A total of 281 patients were included, comprising 84 VH, 86 LH, and 111 AH cases. Significant differences were observed in age, with VH patients being older (mean 67.2±10.4 years) compared to those in LH (53.4±8.4 years) and AH (56.8±10.4 years) groups (p<0.001). BMI was slightly higher in the AH group ($26.8\pm 4.8\;\mathrm{kg}/m^{2}$) compared to VH (25.1±3.2 kg/m${}^{2}$) and LH ($25.5\pm 5.2\;\mathrm{kg}/m^{2}$), with a statistically significant difference (p=0.014). ASA physical status classification did not differ significantly among groups (p=0.278), with ASA class II being predominant in all three. Uterine weight differed significantly among the surgical approaches (p<0.001). The VH group had the lowest median uterine weight (41.2 g; IQR 31.0–65.5), followed by the LH group (156.0 g; IQR 95.8–250.3), and AH group (289.0 g; IQR 76.3–684.1), reflecting differing surgical indications. Overall, the most frequent indication for VH was pelvic organ prolapse (approximately 60% of cases), while uterine fibromatosis was the first indication for LH (30%) and AH (55%).

Operative and recovery characteristics are reported in Table 2. Operative time was shortest for VH (73.5±20.0 min), significantly less than both LH (117.3±48.7 min; p<0.001) and AH (108.3±37.4 min; p<0.001). Estimated blood loss was also significantly lower in VH (85.8±44.3 mL) compared to AH (139.4±151.1mL;p=0.002) and LH (118.5±85.8mL;p=0.022), while no significant difference was found between AH and LH.

**Table 2:** Perioperative and recovery characteristics stratified by route of hysterectomy.

Variables	VH	LH	AH	P-value
Operative time (min)	73.46±19.97	117.29±48.65	108.33±37.39	VH vs. LH <0.001
				VH vs. AH <0.001
				LH vs. AH 0.99
Est. blood loss (mL)	85.82±44.29	118.52±85.83	139.42±151.11	VH vs. LH 0.022
				VH vs. AH 0.002
				LH vs. AH 0.99
Days to discharge	2.63±0.74	2.86±1.0	3.38±1.01	VH vs. LH 0.38
				VH vs. AH <0.001
				LH vs. AH <0.001
Painkiller doses	1.98±1.79	1.88±1.67	2.63±2.18	VH vs. LH 0.99
				VH vs. AH 0.37
				LH vs. AH 0.22
First bowel gas (days)	1.04±0.19	1.45±0.57	1.53±0.75	VH vs. LH <0.001
				VH vs. AH <0.001
				LH vs. AH 0.99
Complications, n/N (%)	4/88 (4.54)	4/90 (4.44)	6/117 (5.13)	0.96
Clavien-Dindo grade I	1 (25.0)	0 (0.0)	0 (0.0)	
Clavien-Dindo grade II	3 (75.0)	1 (25.0)	3 (50.0)	
Clavien-Dindo grade IIIa	0 (0.0)	2 (50.0)	0 (0.0)	
Clavien-Dindo grade IIIb	0 (0.0)	1 (25.0)	3 (50.0)	
Clavien-Dindo grade distribution among complications				0.13

AH, abdominal hysterectomy; LH, laparoscopic hysterectomy; VH, vaginal hysterectomy.

Data are presented as mean ± standard deviation for continuous variables and n (%) for categorical variables. For continuous variables, overall group differences were first assessed using the Kruskal-Wallis test; when significant, post-hoc pairwise comparisons were performed using Mann-Whitney U tests with Bonferroni correction. Complications were calculated among all eligible patients before exclusion of complicated postoperative courses from the primary analysis. Percentages in the complication-rate row refer to the total number of eligible patients in each surgical-route group before exclusion. Percentages in the Clavien-Dindo rows refer to the number of complications within each surgical-route group. The P-value in the complication-rate row refers to Fisher’s exact test comparing overall complication rates across surgical routes. The P-value in the Clavien-Dindo grade distribution row refers to Fisher’s exact test comparing the distribution of complication grades across surgical routes; this comparison should be interpreted cautiously because of the small number of events.

Time to first bowel gas passage was significantly shorter in VH (1.04±0.19 days) compared to both LH (1.45±0.57 days; p<0.001) and AH (1.53±0.75 days; p<0.001). Hospital stay for VH (2.63±0.74 days) was similar to that following LH (2.86±1.0 days; p=0.38) and shorter than the one following AH (3.38±1.01 days; p<0.001). The number of postoperative analgesic doses did not differ significantly across groups.

Baseline laboratory values for Neu, Hb, and WBC were comparable among groups prior to surgery (all p>0.05), supporting the homogeneity of systemic inflammation at baseline (see Table 1).

Following surgery, however, significant inflammatory differences emerged (see Table 3). Postoperative CRP levels were markedly higher in AH patients at Days 1, 2, and 3 (all p<0.001), as illustrated in Table 3. On Day 1, median CRP was 3.3 mg/dL (IQR 2.1–4.9) in AH compared to 1.3 mg/dL (IQR 0.9–2.0) in VH and 1.3 mg/dL (IQR 0.8–2.2) in LH (VH vs. AH, p<0.001). Similar patterns persisted on Day 2 (AH 6.0 mg/dL vs. VH 2.2 mg/dL, p<0.001) and Day 3 (AH 4.8 mg/dL vs. VH 2.4 mg/dL, p<0.001).

**Table 3:** Inflammatory and hematologic markers stratified by surgical approach.

Variables	VH	LH	AH	P-value
Postoperative markers				
Hb Day 1 (g/dL)	11.9 (11.0–12.8)	11.8 (11.0–12.6)	11.7 (10.6–12.6)	0.23
Hb Drop (g/dL)	1.5 (0.9–1.9)	1.4 (0.9–2.2)	1.4 (0.9–2.2)	0.94
CRP (mg/dL)				
Day 1	1.3 (0.9–2.0)	1.3 (0.8–2.2)	3.3 (2.1–4.9)	VH vs. LH 0.99
				VH vs. AH <0.001
				LH vs. AH <0.001
Day 2	2.2 (1.1–3.3)	2.7 (1.5–4.3)	6.0 (3.7–9.4)	VH vs. LH 0.13
				VH vs. AH <0.001
				LH vs. AH <0.001
Day 3	2.4 (1.1–4.7)	2.2 (1.3–4.3)	4.8 (3.1–8.0)	VH vs. LH 0.99
				VH vs. AH <0.001
				LH vs. AH <0.001
Neu ($\times 10^{3}/\text{μ}L$)				
Day 1	9.1 (7.7–10.8)	8.0 (6.9–9.8)	10.5 (7.7–12.2)	VH vs. LH 0.118
				VH vs. AH 0.024
				LH vs. AH <0.001
Day 2	6.0 (4.9–7.3)	4.5 (3.6–5.6)	6.1 (4.9–7.6)	VH vs. LH <0.001
				VH vs. AH 0.99
				LH vs. AH <0.001
Day 3	4.7 (3.7–5.8)	3.9 (3.2–5.6)	4.7 (3.9–6.0)	VH vs. LH 0.24
				VH vs. AH 0.99
				LH vs. AH 0.098
WBC ($\times 10^{3}/\text{μ}L$)				
Day 1	11.7 (10.3–13.4)	10.3 (9.1–12.3)	12.7 (10.5–14.5)	VH vs. LH 0.009
				VH vs. AH 0.39
				LH vs. AH <0.001
Day 2	9.1 (7.7–10.1)	6.9 (6.0–8.6)	8.7 (7.4–10.3)	VH vs. LH <0.001
				VH vs. AH 0.99
				LH vs. AH <0.001
Day 3	7.7 (6.8–9.3)	6.8 (5.5–8.1)	7.5 (6.3–8.8)	VH vs. LH 0.058
				VH vs. AH 0.83
				LH vs. AH 0.15

Data are presented as median (interquartile range). Overall group differences were first assessed using the Kruskal-Wallis test; when significant, post-hoc pairwise comparisons were performed using Mann-Whitney U tests with Bonferroni correction.

CRP, C-reactive protein; WBC, white blood cells; Neu, neutrophils; Hb, hemoglobin; AH, abdominal hysterectomy; LH, laparoscopic hysterectomy; VH, vaginal hysterectomy.

Neu and WBC counts also peaked at higher levels in the AH group, especially on postoperative Days 1 and 2. For instance, on Day 1, median Neu count in AH was $10.5\times 10^{3}/\text{μ}L$ compared to $9.1\times 10^{3}/\text{μ}L$ in VH and $8.0\times 10^{3}/\text{μ}L$ in LH. On Day 2, a significant difference was noted between VH and LH (6.0 vs. $4.5\times 10^{3}/\text{μ}L$; p<0.001), whereas VH and AH were comparable (p=0.99). WBC values followed similar trends. The postoperative trajectories of CRP levels, neutrophil counts, and white blood cell counts across the three surgical approaches are illustrated in Figure 2.

**Figure 2:**
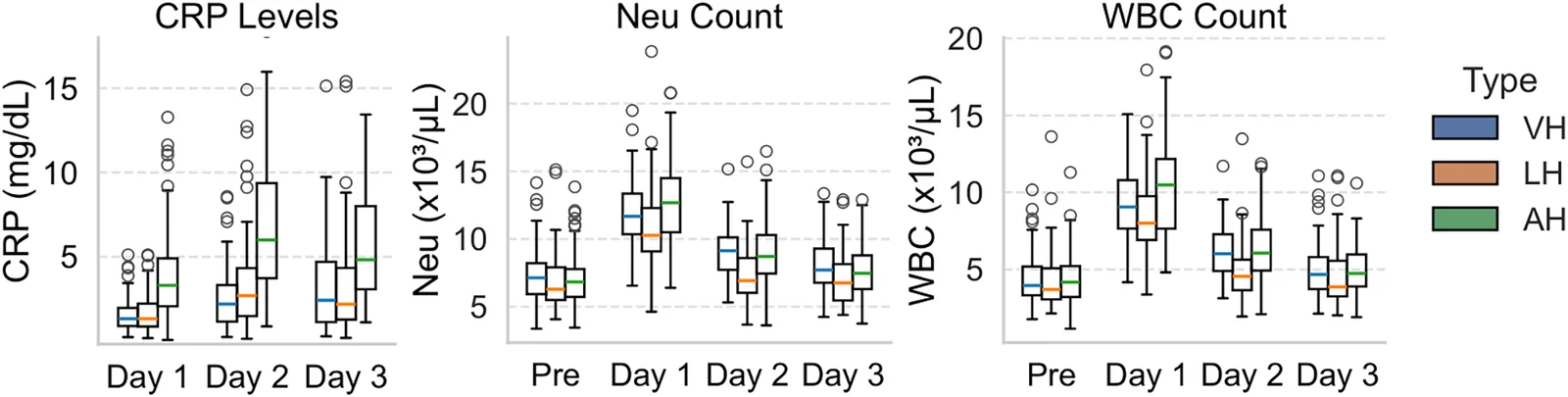
Boxplots of postoperative inflammatory and hematologic markers stratified by surgical approach for C-reactive protein, neutrophils and white blood cell count. CRP values are shown for postoperative Days 1, 2, and 3, as preoperative CRP was not routinely measured. Neutrophil and white blood cell counts are shown from preoperative baseline to postoperative Days 1, 2, and 3. The three surgical routes—VH, LH, and AH—are distinguished by different median-line colors. AH, abdominal hysterectomy; CRP, C-reactive protein; LH, laparoscopic hysterectomy; Neu, neutrophils; VH, vaginal hysterectomy; WBC, white blood cells.

Hemoglobin drop was comparable among all groups, with median decreases of 1.5 g/dL in VH, 1.4 g/dL in LH, and 1.4 g/dL in AH (p=0.94), supporting the notion that blood loss alone is not a major driver of inflammatory differences.

Multivariable regression was used to assess the independent association between surgical approach and postoperative inflammatory markers, adjusting for age, BMI, ASA physical status, surgical indication, and previous abdominal surgery, with VH as the reference category (see Tables 4–7). Surgical route and indication were strongly collinear (VIFs were 9.7 for LH and 10.2 for AH, and 8.1 for the prolapse indication), reflecting the near-complete overlap between VH and the prolapse indication; the remaining covariates showed no collinearity (VIF<1.6). In the day-specific models, AH was independently associated with higher CRP than VH on postoperative Day 2 (log-scale β=0.90,95% CI 0.28 to 1.52, p=0.004), with a consistent but non-significant direction on Day 1 (β=0.12, 95% CI −0.41 to 0.66, p=0.65) and Day 3 (β=0.66, 95% CI −0.03 to 1.35, p=0.062). LH was associated with lower CRP than VH on postoperative Day 1 (β=−0.73, 95% CI −1.29 to −0.16, p=0.012), whereas no significant differences were observed on Days 2 or 3. Overall, no sustained difference between VH and LH was identified across the postoperative trajectory. Given the collinearity between route and indication, these route coefficients are difficult to interpret in isolation; we therefore relied on the mixed-effects and stratified analyses described below. Previous abdominal surgery was associated with lower Day 1 CRP (β=−0.33,p=0.010), and BMI with higher CRP on Days 1 and, at trend level, 3. The linear mixed-effects model, which used all available observations and accounted for within-patient correlation, confirmed that postoperative CRP trajectories differed significantly by surgical route (likelihood-ratio test for the route by day interaction: $\chi^{2}=11.66$, df=4,p=0.020). AH was independently associated with higher CRP overall (β=0.59, 95% CI 0.08 to 1.11, p=0.023), and the route by day interaction indicated that the excess CRP in the AH group was maximal at Day 2 (AH-by-Day 2 interaction β=0.27, p=0.002) and had converged by Day 3. To estimate the route effect free of indication confounding, we restricted the analysis to patients operated for uterine fibromatosis, in whom LH and AH could be compared directly. In this subgroup, AH was independently associated with higher CRP than LH on all three postoperative days (log-scale β=1.05, p<0.001 on Day 1; β=1.07, p<0.001 on Day 2; β=0.98, p=0.006 on Day 3), adjusting for age, BMI, ASA, and uterine weight (see Table 7). This effect was unchanged when uterine weight was omitted and the sample enlarged accordingly (Day 1 β=0.91; Day 2 β=0.93; Day 3 β=0.75; all p≤0.004), and the fibromatosis mixed-effects model showed a stable abdominal-vs.-laparoscopic elevation across days without a significant route by day interaction. For neutrophil and white blood cell counts, no surgical route was independently associated with marker levels at any postoperative day after multivariable adjustment (all p>0.05; Tables 5, 6), indicating that the unadjusted between-route differences in these markers were attenuated once patient-level factors were accounted for. BMI was independently associated with higher neutrophil and WBC counts at later time points, and age with higher counts on Day 2. All models explained a modest proportion of variance (adjusted $R^{2}$ 0.22–0.31 for CRP and 0.06–0.17 for neutrophil and WBC counts).

**Table 4:** Multivariable linear regression of postoperative log-CRP (primary models; VH reference).

Variable	Day 1 (β; 95% CI; p)	Day 2 (β; 95% CI; p)	Day 3 (β; 95% CI; p)
Route: LH vs. VH	−0.73 (−1.29,−0.16); 0.012	0.03 (−0.61,0.68); 0.926	−0.11 (−0.84,0.62); 0.773
Route: AH vs. VH	0.12 (−0.41,0.66); 0.648	0.90 (0.28,1.52); 0.004	0.66 (−0.03,1.35); 0.062
Indication: prolapse	−0.71 (−1.24,−0.17); 0.010	−0.24 (−0.86,0.38); 0.445	−0.21 (−0.88,0.46); 0.537
Prior abd. surgery	−0.33 (−0.59,−0.08); 0.010	−0.19 (−0.46,0.09); 0.182	−0.23 (−0.58,0.12); 0.202
Age	−0.004 (−0.013,0.004); 0.309	0.004 (−0.006,0.014); 0.420	0.011 (−0.001,0.023); 0.074
BMI	0.022 (0.003,0.041); 0.027	0.017 (−0.004,0.038); 0.122	0.025 (−0.001,0.052); 0.056
ASA	−0.21 (−0.43,0.01); 0.066	−0.19 (−0.44,0.05); 0.121	−0.03 (−0.37,0.30); 0.838
Intercept	1.43 (0.48,2.38); 0.003	0.80 (−0.25,1.85); 0.134	−0.11 (−1.39,1.16); 0.861
n/adjusted $R^{2}$	266/0.303	259/0.314	178/0.219

Uterine weight excluded owing to non-random missingness; see Table 7 for the fibromatosis-restricted models that include it.

Coefficients are on the natural-log scale (outcome: ln(CRP)). Reference categories: VH, non-prolapse indication, no previous abdominal surgery. CI, confidence interval.

**Table 5:** Multivariable linear regression of postoperative neutrophil count ($\times 10^{3}/\text{μ}L$; VH reference).

Variable	Day 1 (β; 95% CI; p)	Day 2 (β; 95% CI; p)	Day 3 (β; 95% CI; p)
Route: LH vs. VH	−0.28 (−2.50,1.95); 0.807	−1.38 (−2.87,0.12); 0.072	−0.84 (−2.49,0.80); 0.313
Route: AH vs. VH	1.44 (−0.66,3.55); 0.178	0.03 (−1.39,1.45); 0.966	−0.71 (−2.27,0.85); 0.370
Indication: prolapse	0.37 (−1.73,2.48); 0.727	−0.88 (−2.30,0.54); 0.224	−0.62 (−2.14,0.90); 0.421
Prior abd. surgery	0.71 (−0.29,1.71); 0.162	0.01 (−0.67,0.68); 0.989	−0.16 (−0.95,0.64); 0.698
Age	0.003 (−0.031,0.037); 0.856	0.051 (0.028,0.074); <0.001	0.026 (−0.000,0.053); 0.054
BMI	0.084 (0.008,0.160); 0.030	0.037 (−0.015,0.089); 0.162	0.075 (0.016,0.133); 0.013
ASA	0.23 (−0.65,1.12); 0.602	−0.32 (−0.92,0.29); 0.304	0.40 (−0.33,1.13); 0.278
Intercept	5.40 (1.66,9.15); 0.005	3.23 (0.70,5.75); 0.013	1.43 (−1.43,4.30); 0.325
n/adjusted $R^{2}$	268/0.081	264/0.167	183/0.056

Coefficients represent absolute differences in postoperative neutrophil count ($\times 10^{3}/\text{μ}L$). Reference categories: VH, non-prolapse indication, no previous abdominal surgery. CI, confidence interval.

**Table 6:** Multivariable linear regression of postoperative white blood cell count ($\times 10^{3}/\text{μ}L$; VH reference).

Variable	Day 1 (β; 95% CI; p)	Day 2 (β; 95% CI; p)	Day 3 (β; 95% CI; p)
Route: LH vs. VH	−1.25 (−3.55,1.04); 0.284	−1.56 (−3.30,0.18); 0.078	−1.08 (−2.88,0.71); 0.236
Route: AH vs. VH	0.32 (−1.86,2.50); 0.774	−0.21 (−1.86,1.44); 0.803	−0.82 (−2.53,0.88); 0.342
Indication: prolapse	−0.23 (−2.40,1.95); 0.839	−0.74 (−2.39,0.91); 0.377	−0.37 (−2.03,1.29); 0.664
Prior abd. surgery	0.57 (−0.47,1.60); 0.280	−0.13 (−0.91,0.66); 0.749	−0.005 (−0.88,0.87); 0.991
Age	−0.006 (−0.042,0.029); 0.724	0.050 (0.023,0.077); <0.001	0.032 (0.003,0.061); 0.031
BMI	0.103 (0.024,0.182); 0.011	0.058 (−0.002,0.118); 0.060	0.119 (0.055,0.183); <0.001
ASA	0.10 (−0.81,1.01); 0.828	−0.29 (−0.99,0.41); 0.416	0.26 (−0.53,1.06); 0.515
Intercept	9.27 (5.39,13.14); <0.001	5.59 (2.65,8.52); <0.001	2.84 (−0.29,5.97); 0.075
n/adjusted $R^{2}$	268/0.062	264/0.140	183/0.112

Coefficients represent absolute differences in postoperative white blood cell count ($\times 10^{3}/\text{μ}L$). Reference categories: VH, non-prolapse indication, no previous abdominal surgery. CI, confidence interval.

**Table 7:** Multivariable linear regression of postoperative log-CRP restricted to patients operated for uterine fibromatosis (LH reference; includes uterine weight).

Variable	Day 1 (β; 95% CI; p)	Day 2 (β; 95% CI; p)	Day 3 (β; 95% CI; p)
Route: AH vs. LH	1.05 (0.59,1.52); <0.001	1.07 (0.60,1.54); <0.001	0.98 (0.30,1.65); 0.006
Age	−0.001 (−0.025,0.023); 0.942	0.001 (−0.024,0.025); 0.952	0.018 (−0.012,0.047); 0.231
BMI	0.019 (−0.016,0.054); 0.272	0.012 (−0.024,0.047); 0.515	0.028 (−0.012,0.067); 0.164
ASA	−0.17 (−0.62,0.28); 0.445	−0.08 (−0.54,0.38); 0.723	0.09 (−0.41,0.58); 0.726
ln(uterine weight)	0.015 (−0.151,0.180); 0.859	0.074 (−0.093,0.242); 0.377	−0.007 (−0.191,0.178); 0.943
Intercept	−0.04 (−2.16,2.07); 0.969	0.09 (−2.05,2.23); 0.933	−1.28 (−3.73,1.17); 0.297
n/adjusted $R^{2}$	55/0.273	55/0.283	44/0.196

Coefficients on the natural-log scale. Reference category: LH; VH was not performed for fibromatosis. Sensitivity models omitting uterine weight (larger n): AH-vs-LH β=0.91 (Day 1), 0.93 (Day 2), 0.75 (Day 3), all p≤0.004.

## 4 Discussion

The primary objective of our study was to evaluate the postoperative inflammatory response in relation to the surgical approach used for hysterectomy. This retrospective analysis demonstrates that the route of hysterectomy significantly influences the magnitude of the postoperative systemic inflammatory response. AH was associated with a more intense systemic inflammatory response and slower functional recovery, consistent with its greater invasiveness. After multivariable adjustment, AH remained independently associated with higher postoperative CRP; because surgical route and indication were strongly collinear, this association was most clearly demonstrated within the fibromatosis subgroup, where AH was associated with higher CRP than LH across all postoperative days. No sustained independent differences were observed between VH and LH. However, although significant differences in neutrophil and white blood cell counts were observed at specific postoperative time points in unadjusted analyses, these differences were largely attenuated after multivariable adjustment. This finding suggests that Neu and WBC counts are more sensitive to patient-related factors and perioperative variability than to surgical access alone. In contrast, both VH and LH were associated with a milder postoperative inflammatory response, supporting their minimally invasive nature. Notably, VH demonstrated a postoperative inflammatory profile largely comparable to LH, particularly with respect to CRP kinetics, while conferring additional clinical advantages such as faster functional recovery. Although transient differences in neutrophil and WBC counts were observed at specific postoperative time points, these were not sustained after multivariable adjustment. Taken together, these findings support the use of minimally invasive techniques and suggest that, when clinically appropriate and technically feasible, VH is associated with excellent perioperative outcomes and a systemic inflammatory response broadly comparable to that observed after LH.

An important concern in observational comparisons between surgical approaches is whether observed differences reflect the surgical technique itself or rather surgeon-related factors and selection bias driven by surgical indications. In the present study, all procedures were performed by a limited number of surgeons within the same surgical team, each experienced in all three approaches, thereby reducing the likelihood that differences in outcome were primarily attributable to operator expertise. Moreover, surgical access was determined according to preoperative anatomical and clinical criteria rather than individual surgeon preference, and major determinants of case complexity, including surgical indication and previous abdominal surgery, were accounted for in multivariable analyses, and the route effect was additionally examined within a single-indication subgroup to reduce confounding by indication.

The demographic heterogeneity observed among the study groups is largely attributable to the retrospective nature of the analysis. In particular, the unequal distribution of surgical indications across approaches, with pelvic organ prolapse prevailing in VH and fibromatosis in the laparoscopic and abdominal groups, reflects different anatomical and clinical scenarios rather than surgeon-dependent allocation. The older age of patients who underwent VH reflects the primary indication for this approach, namely pelvic organ prolapse. As for the observed differences in BMI, although statistically significant, they were modest in absolute terms. From the multivariate analysis, BMI emerged as the only preoperative independent predictor of postoperative inflammatory burden at later times, particularly for CRP, neutrophil, and WBC levels on postoperative Day 3, underscoring its contributing role beyond the surgical approach. Uterine weight reflected the predominant indications for LH and AH, particularly uterine fibromatosis. Despite the substantial differences observed across surgical approaches, uterine weight did not emerge as an independent determinant of postoperative inflammatory response in the fibromatosis-restricted analysis. In women with uteri weighing more than 500 g, a later comparative study found no significant differences in estimated blood loss or hemoglobin decline between VH and LH (11).

The clinical advantages of VH and LH over AH are already well established; therefore, the contribution of the present study is not to redefine the recommended hierarchy of surgical routes, but to provide a complementary biological assessment of surgical trauma. Specifically, we directly compared serial postoperative CRP, WBC, and Neu trajectories across VH, LH, and AH within a single-center cohort managed according to standardized perioperative protocols. By excluding complicated postoperative courses and incorporating multivariable, mixed-effects, and indication-restricted analyses, we sought to distinguish the inflammatory effect associated with surgical access from the effects of postoperative complications and major clinical confounders. Our findings demonstrate a greater CRP burden after AH, whereas no sustained difference in inflammatory trajectories was identified between VH and LH. At the same time, VH retained advantages over LH in operative time, blood loss, and bowel-function recovery. To our knowledge, only one other study in the literature has adopted a similar design, comparing three surgical approaches with the aim of analyzing cytokine-mediated inflammatory responses. However, that study limited its inflammatory assessment to the neutrophil-to-lymphocyte ratio (NLR), without reporting postoperative CRP levels, a parameter more widely used in studies comparing two techniques. The authors reported a greater inflammatory variation following VH, comparable to that observed after laparotomy, at least during the first postoperative day, a finding that diverges from our results. Nonetheless, a closer examination of their data reveals that estimated blood loss in the VH group was intermediate between that of LH and laparotomy, potentially indicating more technically demanding or complex procedures, which may partly explain the observed increase in inflammatory response (12). The majority of studies investigating the postoperative inflammatory response have focused on comparisons between AH and LH. These studies support our findings, consistently demonstrating markedly higher CRP levels following laparotomy compared to laparoscopy, indicating a less pronounced inflammatory response with the minimally invasive technique. Most of these investigations also included interleukin-6 (IL-6), which, similar to CRP, was significantly elevated in the AH group. Some additionally assessed cortisol, although findings for this marker were less consistent (13–16). In our cohort, this trend was also evident in CRP levels, which on postoperative Day 1 were 3.3 mg/dL in the AH group vs. 1.3 mg/dL in both VH and LH groups, with similar disparities persisting on Days 2 and 3. Neu and WBC counts followed a consistent pattern, with peak values in AH on Days 1–2, confirming the sustained inflammatory burden associated with the open approach. However, their reduced independent association with surgical route after multivariable adjustment suggests that CRP may represent a more robust and access-specific biomarker of surgical tissue trauma in uncomplicated hysterectomy. A later prospective study comparing AH, VH, and LH in women with benign uterine disease likewise found higher postoperative interleukin-6 (IL-6) and creatine phosphokinase (CPK) levels after AH than after either minimally invasive approach (9). More recent randomized evidence comparing robotic-assisted laparoscopic with abdominal hysterectomy also demonstrated lower IL-6, white blood cell, and cortisol responses with the minimally invasive approach (17). These findings broadly support our observation of a greater postoperative inflammatory burden after AH. Comparatively fewer studies have directly examined differences in inflammatory response between VH and LH. In the three-route comparison by Oksuzoglu et al., postoperative IL-6 was significantly higher after VH than after LH, whereas CPK did not differ between the two minimally invasive groups (9). In contrast, when comparing the two minimally invasive techniques, LH seems to be favored. In one study that also included LAVH, the reported CRP level on postoperative day three in the VH group was 7.6 mg/dL, whereas in the LH group it was 4.1 mg/dL, values that differ markedly from those observed in our cohort. In our study, CRP values on postoperative Day 3 were 2.4 mg/dL and 2.2 mg/dL for VH and LH, respectively, with a non-significant p-value of 0.99 (18). Therefore, within the context of the available literature, our findings add further evidence suggesting that VH is associated with postoperative inflammatory profiles largely comparable to those observed after LH in uncomplicated cases. This apparent similarity was further supported by multivariable models, which showed no sustained independent differences in postoperative CRP, WBC, or Neu counts between VH and LH across postoperative time points. However, the use of VH for uterine removal offers significant advantages when compared to LH not only intraoperatively, but also during the early postoperative period. In particular, our findings add to the growing body of literature suggesting that VH may be associated with shorter operative times and reduced intraoperative blood loss (3). Moreover, although data regarding postoperative length of stay for the two minimally invasive techniques remain inconsistent, VH appears to be associated with faster return of bowel function and potentially shorter hospital stay (19, 20). Across models, statistically significant associations between surgical approach and postoperative inflammation were observed, yet these explained only a modest proportion of the variability in inflammatory markers. This finding is consistent with the multifactorial nature of postoperative inflammation, which is influenced not only by surgical technique, but also by patient specific factors, perioperative management, and individual biological responses. Given the retrospective design, these findings should be interpreted as associative rather than causal, while still providing clinically meaningful insight into access-related differences in perioperative inflammatory burden.

From a clinical perspective, although absolute CRP values remained within the expected range for uncomplicated surgery, the consistent and independent differences observed between abdominal and minimally invasive approaches across all postoperative days reflect a clinically meaningful variation in tissue injury and recovery kinetics. This is further supported by the parallel differences observed in functional recovery milestones, including bowel function and length of hospitalization, reinforcing the translational relevance of the inflammatory findings.

Although patients with postoperative complications were excluded from the primary inflammatory analysis by study design, complication data were recorded and reported descriptively among all eligible patients. The overall complication rate was low and did not differ meaningfully among surgical routes. Severe complications requiring surgical or interventional management were observed only after AH and LH; however, the number of events was small, and the study was not powered to compare complication rates or safety profiles between approaches. Therefore, complication data should be interpreted cautiously and were not used to support comparative conclusions regarding the safety of one route over another. Their exclusion from the main analysis was intended to isolate inflammatory trajectories after uncomplicated hysterectomy, but this choice may limit generalizability and introduce post-treatment selection bias.

This study presents several limitations inherent to its retrospective observational design. First, the lack of randomization introduces the potential for selection bias, and although baseline characteristics were largely comparable across groups, significant differences in patient age, uterine weight, and BMI were observed. These demographic imbalances may reflect surgical selection criteria but also represent possible confounding factors that could have influenced the perioperative and inflammatory outcomes. In particular, surgical route was strongly collinear with surgical indication, since VH was used almost exclusively for prolapse and fibromatosis occurred only in the laparoscopic and abdominal groups; the individual route coefficients in the full model must therefore be interpreted with caution, and residual confounding by indication cannot be excluded despite adjustment and stratification. In addition, uterine weight was available for 188 of 281 patients and was analysed as a sensitivity covariate rather than in the primary models; reassuringly, the fibromatosis-restricted findings were consistent with and without adjustment for uterine weight. Secondly, this was a single-center study, with all surgeries performed by a limited number of surgeons using standardized protocols. While this enhances internal consistency and reduces variability in surgical skill or perioperative management, it may also limit external validity, as outcomes could differ in other clinical settings with different surgical expertise, patient populations, or postoperative care protocols. Thirdly, we note that the inflammatory assessment was limited to routinely available laboratory markers (CRP, WBC, and Neu). In addition, preoperative CRP values were not available because CRP assessment is not part of the standard preoperative laboratory evaluation for asymptomatic patients undergoing elective benign hysterectomy at our institution. Therefore, CRP could not be analyzed as a change from baseline, and postoperative CRP values should be interpreted as postoperative levels rather than true perioperative variations. Other inflammatory or stress-related biomarkers, such as interleukin-6, cortisol, or procalcitonin, were not systematically collected in routine clinical practice during the study period and were therefore unavailable for analysis. Although the inclusion of these markers could have provided additional mechanistic insight, CRP, WBC, and Neu count are well-validated indicators of postoperative systemic inflammation and are widely used in clinical settings, supporting the robustness and clinical relevance of the present findings. Furthermore, the modest explanatory power of multivariate models underscores the presence of unmeasured factors influencing postoperative inflammation, which could not be fully captured within the constraints of a retrospective design. In addition, patients who experienced postoperative complications were excluded from the main analysis. While this choice aligns with the primary objective of characterizing the inflammatory response following uncomplicated hysterectomy, it may limit the generalizability of the findings to more complex postoperative courses. Including complicated cases would have introduced additional clinical heterogeneity related to the type and severity of complications and their management, potentially confounding the interpretation of access-related inflammatory patterns.

Future studies on larger cohorts should specifically explore inflammatory trajectories in patients who develop postoperative complications and perform sensitivity analyses incorporating these cases.

## 5 Conclusions

In patients undergoing uncomplicated total hysterectomy for benign disease, AH was associated with higher postoperative CRP levels, higher blood loss, delayed recovery milestones, and longer hospitalization compared with minimally invasive approaches, whereas both AH and LH required longer operative times than VH. Between the two minimally invasive techniques, postoperative inflammatory profiles were broadly comparable, whereas VH was associated with shorter operative time, lower blood loss, and faster bowel-function recovery than LH. These findings suggest that VH represents an effective but underutilized minimally invasive option when clinically appropriate. Overall, the results support the prioritization of minimally invasive techniques when feasible, while recognizing that the retrospective design, indication-related selection bias, and exclusion of complicated postoperative courses require the findings to be interpreted as associations rather than causal effects.

## Data Availability

The datasets presented in this article are not publicly available due to patient privacy considerations, but may be made available by the authors upon reasonable request, subject to applicable ethical and data protection requirements.
